# Genome of Myoviridae Phage Lethe Isolated In Northern Georgia

**DOI:** 10.17912/micropub.biology.001984

**Published:** 2026-04-07

**Authors:** Paige Alexander, Maggie Blair, Emma Boyen, Lavender Bumgarner, Gabrielle Grogan, Ella Lesperance, Alison Kanak

**Affiliations:** 1 University of North Georgia, Dahlonega, GA, US; 2 Biology, University of North Georgia, Dahlonega, GA, US

## Abstract

Lethe, a predicted lytic bacteriophage with myovirus morphology, was isolated using *Mycobacterium smegmatis mc²155* in North Georgia. Lethe has a genome of 155,828 base pairs with 230 predicted ORFs, 34 tRNAs, 1 tmRNA, and 64.70% GC content. Based on gene content, Lethe is assigned to actinobacteriophage cluster C1, sharing up to 99.5% nucleotide identity with members of this cluster.

**Figure 1. Structure of phage particles, plaques, and protein:** A. Transmission electron micrograph (TEM) of Lethe. The TEM was obtained using a J JEOL 2100PLUS (JOEL, Inc., Tokyo, Japan) at the University of Georgia Electron Microscope laboratory. B. Representative plaques produced by Lethe are turbid and 1-5 mm in size. C. Structure prediction of ORF 125 using I-TASSER includes multiple alpha helices (in magenta) and beta sheets (in orange).

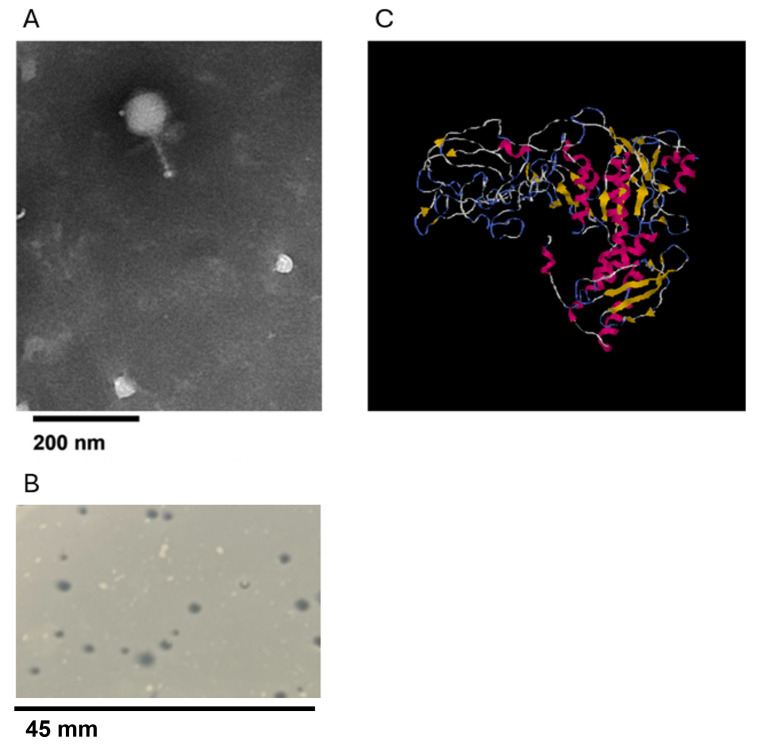



## Description

The study of bacteriophage, viruses that prey on bacterial hosts, is increasingly important due to their use as an alternative to antibiotics. Complementing these efforts is the SEA-PHAGES program that mobilizes thousands of undergraduate students in isolating and characterizing novel bacteriophages (Heller et al, 2024).

Lethe was isolated from an environmental sample collected at Lake Zwerner, which is in northeast downtown Dahlonega, GA (34.55437°N, 83.96678°W). The sample was fine, silt like, watery, light brown soil from the bottom of the lake. After collection, the sample was filtered then suspended in Middlebrook 7H9 liquid medium and the suspension was inoculated with *Mycobacterium smegmatis* mc ^2^ 155 and incubated with shaking overnight at 37°C. The resulting culture was filtered with a 0.22µm filter and the filtrate plated on 7H10 agar with *M. smegmatis* in a standard plaque assay (Zorawik et al., 2024). Plates were incubated at 37°C for 48 hours. After four rounds of picking plaques and plating, a clonal population of Lethe was found to form plaques that are 1-5 mm in diameter, turbid, and rounded with defined edges (Fig 1B). A high titer lysate of Lethe was prepared and used for electron microscopy and genomic DNA extraction. &nbsp;Phosphotungstic acid (1 %) and a parafilm drop method was used to stain and mount the phage sample for negative stain transmission electron microscopy, which revealed Lethe to possess a myovirus morphology with a capsid measuring 69.12 nm in diameter as well as a tail measuring 80.96 nm in length as measured using eleif.net/photomeasure (n=1; Figure 1A ).

Phage genomic DNA was isolated from Lethe's lysate using a Wizard DNA extraction kit (Promega) per standard Science Education Alliance (SEA) protocol instructions. DNA was prepared for sequencing using the NEB Ultra II Library Kit 9 and sequenced using an Illumina NextSeq 1000&nbsp; with XLEAP-P1 reagents. The Illumina shotgun sequencing method with an Illumina NextSeq 1000 (XLEAP-P1 kit) was used yielding 2.5 million 100 base reads that resulted in 1529 coverage of an assembled genome. The genome is 155828 bps long with 64.7% GC content and has circularly permuted ends. Lethe is a member of the C1 cluster of Actinobacteriophage due to gene content similarity of at least 35% to phages in the Actinobacteriophage database, phagesdb.org (Russell & Hatfull, 2017; Pope et al, 2017).

Lethe's genome was automatically annotated using Glimmer v3.02 (Delcher et al., 2007) and GeneMark v2.5p (Besemer & Borodovsky, 2005) through PECAAN v20240320 (Rinehart CA, Gaffney BL, et al. 2016). and DNA Master V5.23.6 (Pope & Jacobs-Sera, 2018). The annotation was then manually refined using Starterator v557, Phamerator v557 (Cresawn et al., 2011) using the Actino_draft database, NCBI BLASTp v2.15.0 (Altschul et al., 1990) searches against the Actinobacteriophage and NCBI non-redundant databases, HHPred v2.08 (Zimmermann et al., 2018) searches against the PDB_mmCIF70, Pfam-v.36, NCBI Conserved Domains databases, tRNAscanSE v2.0 (Chan, et al 2021), Aragorn (Laslett and Canback 2004 **)** TMHMM v.1.0.24 (Chen et al., 2003), TOPCONS v2.0, and I-TASSER (Zhang, 2008). All software was run on the default parameters. Where applicable, hits with E values of 10e-10 or less were considered acceptable. Lethe has 230 predicted open reading frames. Putative functions were predicted for 51 of these, including a predicted tail sheath protein (gp125). Using I-TASSER ( Figure 1C ), the multiple alpha helices and beta sheets that are key to the structure and functioning of the contractile myoviurs tail can be observed (Akysukk et al., 2009). Lethe also encodes 34 tRNAs and one tmRNA. All but five genes are transcribed unidirectionally. These features are consistent with phages in the C1 cluster, of which there are over 200, to-date. Like other phages in this cluster, Lethe is predicted to be lytic (Hatfull, 2012) and does not encode the immunity repressor of cluster A phages. This has been observed for a small but growing subset of C1 phages and is predicted to provide superinfection immunity to cluster A during lytic infection (Pope et al., 2011). A blastn search of the Lethe genomic sequence against the Actinobacteriophage database reveals C1 phage Iota (Genbank accession no. MK359330.1) as the most genetically similar phage, with 99% percent nucleotide identity across a majority of the genome.&nbsp;

Nucleotide sequence accession numbers

Lethe is available at GenBank with Accession No. PV876977 and Sequence Read Archive (SRA) No. SRX29990114 .

## References

[R1] Aksyuk AA, Leiman PG, Kurochkina LP, Shneider MM, Kostyuchenko VA, Mesyanzhinov VV, Rossmann MG (2009). The tail sheath structure of bacteriophage T4: a molecular machine for infecting bacteria.. EMBO J.

[R2] Altschul Stephen F., Gish Warren, Miller Webb, Myers Eugene W., Lipman David J. (1990). Basic local alignment search tool. Journal of Molecular Biology.

[R3] Besemer J., Borodovsky M. (2005). GeneMark: web software for gene finding in prokaryotes, eukaryotes and viruses. Nucleic Acids Research.

[R4] Chan Patricia P, Lin Brian Y, Mak Allysia J, Lowe Todd M (2021). tRNAscan-SE 2.0: improved detection and functional classification of transfer RNA genes. Nucleic Acids Research.

[R5] Chen Yunjia, Yu Peng, Luo Jingchu, Jiang Ying (2003). Secreted protein prediction system combining CJ-SPHMM, TMHMM, and PSORT. Mammalian Genome.

[R6] Cresawn Steven G, Bogel Matt, Day Nathan, Jacobs-Sera Deborah, Hendrix Roger W, Hatfull Graham F (2011). Phamerator: a bioinformatic tool for comparative bacteriophage genomics. BMC Bioinformatics.

[R7] Delcher Arthur L., Bratke Kirsten A., Powers Edwin C., Salzberg Steven L. (2007). Identifying bacterial genes and endosymbiont DNA with Glimmer. Bioinformatics.

[R8] Heller Danielle M., Sivanathan Viknesh, Asai David J., Hatfull Graham F. (2024). SEA-PHAGES and SEA-GENES: Advancing Virology and Science Education. Annual Review of Virology.

[R9] Laslett D. (2004). ARAGORN, a program to detect tRNA genes and tmRNA genes in nucleotide sequences. Nucleic Acids Research.

[R10] Pope WH, Jacobs-Sera D, Russell DA, Peebles CL, Al-Atrache Z, Alcoser TA, Alexander LM, Alfano MB, Alford ST, Amy NE, Anderson MD, Anderson AG, Ang AA, Ares M Jr, Barber AJ, Barker LP, Barrett JM, Barshop WD, Bauerle CM, Bayles IM, Belfield KL, Best AA, Borjon A Jr, Bowman CA, Boyer CA, Bradley KW, Bradley VA, Broadway LN, Budwal K, Busby KN, Campbell IW, Campbell AM, Carey A, Caruso SM, Chew RD, Cockburn CL, Cohen LB, Corajod JM, Cresawn SG, Davis KR, Deng L, Denver DR, Dixon BR, Ekram S, Elgin SC, Engelsen AE, English BE, Erb ML, Estrada C, Filliger LZ, Findley AM, Forbes L, Forsyth MH, Fox TM, Fritz MJ, Garcia R, George ZD, Georges AE, Gissendanner CR, Goff S, Goldstein R, Gordon KC, Green RD, Guerra SL, Guiney-Olsen KR, Guiza BG, Haghighat L, Hagopian GV, Harmon CJ, Harmson JS, Hartzog GA, Harvey SE, He S, He KJ, Healy KE, Higinbotham ER, Hildebrandt EN, Ho JH, Hogan GM, Hohenstein VG, Holz NA, Huang VJ, Hufford EL, Hynes PM, Jackson AS, Jansen EC, Jarvik J, Jasinto PG, Jordan TC, Kasza T, Katelyn MA, Kelsey JS, Kerrigan LA, Khaw D, Kim J, Knutter JZ, Ko CC, Larkin GV, Laroche JR, Latif A, Leuba KD, Leuba SI, Lewis LO, Loesser-Casey KE, Long CA, Lopez AJ, Lowery N, Lu TQ, Mac V, Masters IR, McCloud JJ, McDonough MJ, Medenbach AJ, Menon A, Miller R, Morgan BK, Ng PC, Nguyen E, Nguyen KT, Nguyen ET, Nicholson KM, Parnell LA, Peirce CE, Perz AM, Peterson LJ, Pferdehirt RE, Philip SV, Pogliano K, Pogliano J, Polley T, Puopolo EJ, Rabinowitz HS, Resiss MJ, Rhyan CN, Robinson YM, Rodriguez LL, Rose AC, Rubin JD, Ruby JA, Saha MS, Sandoz JW, Savitskaya J, Schipper DJ, Schnitzler CE, Schott AR, Segal JB, Shaffer CD, Sheldon KE, Shepard EM, Shepardson JW, Shroff MK, Simmons JM, Simms EF, Simpson BM, Sinclair KM, Sjoholm RL, Slette IJ, Spaulding BC, Straub CL, Stukey J, Sughrue T, Tang TY, Tatyana LM, Taylor SB, Taylor BJ, Temple LM, Thompson JV, Tokarz MP, Trapani SE, Troum AP, Tsay J, Tubbs AT, Walton JM, Wang DH, Wang H, Warner JR, Weisser EG, Wendler SC, Weston-Hafer KA, Whelan HM, Williamson KE, Willis AN, Wirtshafter HS, Wong TW, Wu P, Yang Yj, Yee BC, Zaidins DA, Zhang B, Zúniga MY, Hendrix RW, Hatfull GF (2011). Expanding the diversity of mycobacteriophages: insights into genome architecture and evolution.. PLoS One.

[R11] Pope Welkin H., Jacobs-Sera Deborah (2017). Annotation of Bacteriophage Genome Sequences Using DNA Master: An Overview. Methods in Molecular Biology.

[R12] Rinehart, C.A., Gaffney, B.L., Smith, J.R. and Wood, J.D., 2016. PECAAN: bacteriophage evidence collection and annotation network user guide.&nbsp; *Western Kentucky University Bioinformatics and Information Science Center, Bowling Green, KY*

[R13] Russell DA, Hatfull GF (2017). PhagesDB: the actinobacteriophage database.. Bioinformatics.

[R14] Zhang Yang (2008). I-TASSER server for protein 3D structure prediction. BMC Bioinformatics.

[R15] Zimmermann L, Stephens A, Nam SZ, Rau D, Kübler J, Lozajic M, Gabler F, Söding J, Lupas AN, Alva V (2017). A Completely Reimplemented MPI Bioinformatics Toolkit with a New HHpred Server at its Core.. J Mol Biol.

[R16] Zorawik Michelle, Jacobs-Sera Deborah, Freise Amanda C., Reddi Krisanavane, SEA-PHAGES (2024). Isolation of Bacteriophages on Actinobacteria Hosts. Methods in Molecular Biology.

